# Relative predictive value of sociodemographic factors for chronic diseases among *All of Us* participants: a descriptive analysis

**DOI:** 10.1186/s12889-024-17834-1

**Published:** 2024-02-08

**Authors:** Ansley J. Kunnath, Daniel E. Sack, Consuelo H. Wilkins

**Affiliations:** 1 Vanderbilt University Medical Scientist Training Program, Vanderbilt University School of Medicine, Nashville, TN USA; 2 Department of Medicine, Vanderbilt University Medical Center, 2525 West End, Suite 600, Nashville, TN 37203 USA

**Keywords:** Social determinants of health, Chronic diseases, All of Us Research Program

## Abstract

**Background:**

Although sociodemographic characteristics are associated with health disparities, the relative predictive value of different social and demographic factors remains largely unknown. This study aimed to describe the sociodemographic characteristics of *All of Us* participants and evaluate the predictive value of each factor for chronic diseases associated with high morbidity and mortality.

**Methods:**

We performed a cross-sectional analysis using de-identified survey data from the *All of Us* Research Program, which has collected social, demographic, and health information from adults living in the United States since May 2018. Sociodemographic data included self-reported age, sex, gender, sexual orientation, race/ethnicity, income, education, health insurance, primary care provider (PCP) status, and health literacy scores. We analyzed the self-reported prevalence of hypertension, coronary artery disease, any cancer, skin cancer, lung disease, diabetes, obesity, and chronic kidney disease. Finally, we assessed the relative importance of each sociodemographic factor for predicting each chronic disease using the adequacy index for each predictor from logistic regression.

**Results:**

Among the 372,050 participants in this analysis, the median age was 53 years, 59.8% reported female sex, and the most common racial/ethnic categories were White (54.0%), Black (19.9%), and Hispanic/Latino (16.7%). Participants who identified as Asian, Middle Eastern/North African, and White were the most likely to report annual incomes greater than $200,000, advanced degrees, and employer or union insurance, while participants who identified as Black, Hispanic, and Native Hawaiian/Pacific Islander were the most likely to report annual incomes less than $10,000, less than a high school education, and Medicaid insurance. We found that age was most predictive of hypertension, coronary artery disease, any cancer, skin cancer, diabetes, obesity, and chronic kidney disease. Insurance type was most predictive of lung disease. Notably, no two health conditions had the same order of importance for sociodemographic factors.

**Conclusions:**

Age was the best predictor for the assessed chronic diseases, but the relative predictive value of income, education, health insurance, PCP status, race/ethnicity, and sexual orientation was highly variable across health conditions. Identifying the sociodemographic groups with the largest disparities in a specific disease can guide future interventions to promote health equity.

**Supplementary Information:**

The online version contains supplementary material available at 10.1186/s12889-024-17834-1.

## Background

The social determinants of health encompass a wide range of non-medical factors that can influence health outcomes, such as social, economic, cultural, structural, and environmental conditions [1–3]. Studies suggest that clinical care only accounts for 20% of health outcomes, whereas social determinants contribute up to 50% [3, 4]. Identifying risk factors for specific diseases is critical for guiding preventative efforts and advancing health equity. Different sociodemographic groups experience significant disparities in the morbidity and mortality of chronic diseases including hypertension [5–7], coronary artery disease [5, 8–10], cancer [5, 11–16], lung disease [5, 17–19], diabetes [5, 20–22], obesity [5, 23, 24], and chronic kidney disease [25–29]. Despite advances in detection and treatment of chronic diseases, health disparities have widened over time among racial/ethnic groups [5].

The consequences of social determinants are complex and dynamic, such that each may have a unique contribution to different diseases. For example, an analysis of patients from 169 community health centers across the United States found that physical environments (i.e. rural status and Census region) were strongly predictive of hypertension, but less predictive of cardiovascular disease [30]. This study identified health disparities among different sociodemographic groups in a low-income, uninsured population. However, no studies to date have examined the relative contribution of different social factors across health conditions in a nationally representative population.

In this study, we analyze social determinants of health that fall into priority domains established by Healthy People 2023: economic stability (annual income), education access and quality (educational attainment), and healthcare access and quality (insurance type, health literacy, and primary care history) [4]. Information related to the environment [4] and community support [4] domains were unavailable in the *All of Us* dataset at the time of analysis. We also assess sociodemographic factors commonly associated with health disparities, including age, sex, gender, sexual orientation, race, and ethnicity.

First, we describe how various sociodemographic factors (age, sex, gender, sexual orientation, annual income, educational attainment, insurance type, and health literacy) are distributed within self-identified racial and ethnic categories among participants in the *All of Us* Research Program. We then assess the relative contributions of each of these variables on the likelihood of self-reporting hypertension, coronary artery disease, cancer, lung disease, diabetes, obesity, and chronic kidney disease. We hypothesize that the strongest predictors will vary by disease and that a “one-size-fits-all” approach is insufficient for identifying and preventing health disparities.

## Methods

### Study population and data collection

*All of Us*aims to collect health information from over one million people in the United States, with the goal of advancing personalized health care [31]. *All of Us* is therefore uniquely poised to examine social diversity within self-identified racial and ethnic categories and the interactions between different sociodemographic factors and health conditions. The full *All of Us* Research Program protocol has been published previously [32]. Briefly, adults aged 18 years or older living in the United States were consented and enrolled in *All of Us* through a healthcare provider organization or directly through the website (www.allofus.nih.gov). Participants answered baseline demographic questions and had the option to provide additional information on their medical history. Surveys were completed in either English or Spanish, and resulting data were pooled as per the *All of Us* protocol [32].

For this study, we included sociodemographic and health information from collected from *All of Us* participants who were enrolled from May 2017 to January 2022 and completed the Basics Survey. Sociodemographic data and health history were selected based on the health disparities literature [5–29] and the data available in version 6 of *All of Us*. Age, sex, gender, sexual orientation, race, ethnicity, annual income, educational attainment, insurance type, health literacy, and history of seeing a primary care practitioner (PCP) within the last twelve months were included in the analysis. Self-reported health history included hypertension, coronary artery disease, any cancer, skin cancer, lung disease, diabetes, obesity, and chronic kidney disease. Health literacy was assessed via the Brief Health Literacy Screen, and participants were scored from 3–15 using the three questions, with higher scores indicating higher health literacy [33, 34]. Supplemental Table 1 provides details on each question and how each characteristic was coded.

### Data analysis

All data were analyzed directly in the *All of Us* Researcher Workbench with *R Statistical Software*(version 4.2.2) [35]. After data cleaning (Supplemental Tables 1.1 and 1.2), we compared the distributions of age, sex, gender, sexual orientation, annual income, educational attainment, insurance type, health literacy, and recent PCP visit by self-defined racial and ethnic category. We characterized sociodemographic disparities across racial and ethnic categories as these social constructs are tied to important determinants of health that cannot be directly measured in this study, such as racism. We also included participants who skipped or declined to answer a question. As described in Supplemental Table 1.1, participants were asked, “Which categories describe you? Select all that apply. Note, you may select more than one group.” Participants who selected more than one group were coded as “Multiple.” Sub-categories with 20 or fewer participants were excluded for participant privacy [36].

To assess the importance of each social determinant on each health condition, we compared the relative importance of each predictor (ranked from 1–11) based on each predictor’s adequacy index (-2*log-likelihood of each predictor in the model divided by -2*log-likelihood of the full model) in participants who provided baseline self-reported information on a personal history of hypertension, coronary artery disease, any cancer, skin cancer, lung disease, diabetes, obesity, and chronic kidney disease with logistic regression (Table 1) [37, 38]. We used inverse probability weighting to weight each participant who reported clinical conditions (*n* = 141,878) such that the weighted sample (*n* = 378,811) was representative of the full 372,050 participants in *All of Us* version 6 based on the measured social and demographic variables, excluding PCP status due to a large number of version 6 participants who did not fill out the *All of Us*Healthcare Access and Utilization survey [39, 40]. In all models, age and health literacy were modeled as restricted cubic splines with five knots [37, 38]. As health literacy mediates some racial and ethnic disparities in health outcomes and behaviors [41–47], we analyzed the interaction between health literacy and race/ethnicity. As we were interested in the variance explained by each sociodemographic variable, not the specific impact of each category within each social variable, “Skip” and “Prefer Not To Answer” were included as subcategories for categorical variables. Participants with missing or unknown health outcomes in the weighted sample were excluded from the model for that chronic disease. Code is available at www.github.com/ansleykunnath/allofus.

**Table 1:** *All of Us* (version 6) Participant Information

	**Full Dataset**	**Outcomes Reported**	**Weighted Dataset**
**Characteristic**	*N* = 372,050	*N* = 141,878	*N* = 378,811
**Age – Median (IQR)**	52.7 (36.6, 64.7)	56.1 (39.0, 67.3)	52.3 (36.9, 64.6)
**Sex**			
Missing	6,611.0 (1.8%)	4,926.0 (3.5%)	6,588.0 (1.7%)
Prefer Not to Answer	308.0 (0.1%)	43.0 (0.0%)	612.0 (0.2%)
Skip	3,636.0 (1.0%)	859.0 (0.6%)	4,851.4 (1.3%)
Female	222,461.0 (59.8%)	89,541.0 (63.1%)	231,833.9 (61.2%)
Male	138,817.0 (37.3%)	46,468.0 (32.8%)	134,561.2 (35.5%)
None Of These	138.0 (0.0%)	25.0 (0.0%)	299.8 (0.1%)
Intersex	79.0 (0.0%)	16.0 (0.0%)	65.1 (0.0%)
**Gender**			
Prefer Not to Answer	548.0 (0.1%)	93.0 (0.1%)	1,025.2 (0.3%)
Skip	9,596.0 (2.6%)	5,596.0 (3.9%)	10,523.1 (2.8%)
Woman	221,079.0 (59.4%)	88,766.0 (62.6%)	230,755.0 (60.9%)
Man	138,368.0 (37.2%)	46,260.0 (32.6%)	134,092.1 (35.4%)
Multiple	419.0 (0.1%)	227.0 (0.2%)	412.1 (0.1%)
Non-Binary	919.0 (0.2%)	450.0 (0.3%)	965.7 (0.3%)
Transgender	780.0 (0.2%)	338.0 (0.2%)	719.4 (0.2%)
Additional Options	341.0 (0.1%)	148.0 (0.1%)	318.7 (0.1%)
**Sexual Orientation**			
Prefer Not to Answer	4,970.0 (1.3%)	1,009.0 (0.7%)	5,670.5 (1.5%)
Skip	11,054.0 (3.0%)	5,783.0 (4.1%)	13,487.2 (3.6%)
Straight	322,064.0 (86.6%)	121,416.0 (85.6%)	326,444.7 (86.2%)
Bisexual	13,229.0 (3.6%)	5,361.0 (3.8%)	13,024.7 (3.4%)
Gay	8,444.0 (2.3%)	3,752.0 (2.6%)	8,259.4 (2.2%)
None	7,615.0 (2.0%)	2,532.0 (1.8%)	7,391.0 (2.0%)
Lesbian	4,546.0 (1.2%)	1,970.0 (1.4%)	4,399.4 (1.2%)
Multiple	128.0 (0.0%)	55.0 (0.0%)	134.4 (0.0%)
**Race/Ethnicity**			
Asian	13,271.0 (3.6%)	5,069.0 (3.6%)	13,450.5 (3.6%)
Black	74,017.0 (19.9%)	12,472.0 (8.8%)	77,443.3 (20.4%)
Hispanic	62,230.0 (16.7%)	13,309.0 (9.4%)	69,366.1 (18.3%)
MENA	2,829.0 (0.8%)	1,066.0 (0.8%)	2,811.0 (0.7%)
Multiple	1,097.0 (0.3%)	378.0 (0.3%)	1,185.4 (0.3%)
NHPI	593.0 (0.2%)	116.0 (0.1%)	549.1 (0.1%)
White	200,850.0 (54.0%)	101,431.0 (71.5%)	195,711.6 (51.7%)
Prefer Not to Answer	2,354.0 (0.6%)	424.0 (0.3%)	2,867.3 (0.8%)
Skip	10,915.0 (2.9%)	6,387.0 (4.5%)	11,544.3 (3.0%)
None Of These	3,894.0 (1.0%)	1,226.0 (0.9%)	3,882.7 (1.0%)
**Annual Income**			
Prefer Not to Answer	48,348.0 (13.0%)	11,317.0 (8.0%)	48,557.8 (12.8%)
Skip	28,965.0 (7.8%)	8,208.0 (5.8%)	34,650.4 (9.1%)
less 10 k	52,940.0 (14.2%)	8,177.0 (5.8%)	56,656.8 (15.0%)
10 k 25 k	43,364.0 (11.7%)	12,162.0 (8.6%)	44,179.2 (11.7%)
25 k 35 k	26,276.0 (7.1%)	9,339.0 (6.6%)	26,185.1 (6.9%)
35 k 50 k	28,842.0 (7.8%)	12,494.0 (8.8%)	28,247.3 (7.5%)
50 k 75 k	38,241.0 (10.3%)	19,440.0 (13.7%)	37,465.9 (9.9%)
75 k 100 k	29,442.0 (7.9%)	16,193.0 (11.4%)	28,897.6 (7.6%)
100 k 150 k	35,971.0 (9.7%)	20,927.0 (14.7%)	35,285.9 (9.3%)
150 k 200 k	16,709.0 (4.5%)	9,981.0 (7.0%)	16,286.5 (4.3%)
more 200 k	22,952.0 (6.2%)	13,640.0 (9.6%)	22,398.8 (5.9%)
**Education**			
Prefer Not to Answer	2,305.0 (0.6%)	307.0 (0.2%)	2,652.6 (0.7%)
Skip	12,497.0 (3.4%)	5,853.0 (4.1%)	13,800.7 (3.6%)
Never Attended	534.0 (0.1%)	53.0 (0.0%)	770.4 (0.2%)
One Through Four	3,096.0 (0.8%)	404.0 (0.3%)	4,017.8 (1.1%)
Five Through Eight	8,158.0 (2.2%)	1,134.0 (0.8%)	10,143.3 (2.7%)
Nine Through Eleven	22,450.0 (6.0%)	2,511.0 (1.8%)	24,893.4 (6.6%)
Twelve Or GED	70,982.0 (19.1%)	13,613.0 (9.6%)	73,260.4 (19.3%)
College One to Three	92,755.0 (24.9%)	32,605.0 (23.0%)	92,165.0 (24.3%)
College Graduate	81,622.0 (21.9%)	40,067.0 (28.2%)	80,510.8 (21.3%)
Advanced Degree	77,651.0 (20.9%)	45,331.0 (32.0%)	76,597.0 (20.2%)
**Health Insurance**			
Missing	76,213.0 (20.5%)	28,754.0 (20.3%)	76,025.3 (20.1%)
Skip	3,831.0 (1.0%)	581.0 (0.4%)	4,999.9 (1.3%)
Employer Or Union	113,539.0 (30.5%)	56,256.0 (39.7%)	112,795.9 (29.8%)
Medicaid	66,758.0 (17.9%)	11,902.0 (8.4%)	72,188.0 (19.1%)
Multiple	11,413.0 (3.1%)	3,862.0 (2.7%)	11,935.5 (3.2%)
Medicare	58,468.0 (15.7%)	25,387.0 (17.9%)	58,812.7 (15.5%)
Purchased	18,512.0 (5.0%)	8,034.0 (5.7%)	18,381.9 (4.9%)
Other Health Plan	11,526.0 (3.1%)	3,139.0 (2.2%)	11,518.6 (3.0%)
VA	5,279.0 (1.4%)	1,883.0 (1.3%)	4,838.9 (1.3%)
None	2,674.0 (0.7%)	291.0 (0.2%)	3,554.4 (0.9%)
Military	3,837.0 (1.0%)	1,789.0 (1.3%)	3,760.2 (1.0%)
**Medical Form Confidence**			
Skip	14,613.0 (3.9%)	851.0 (0.6%)	17,607.8 (4.6%)
Not At All	7,924.0 (2.1%)	897.0 (0.6%)	9,593.7 (2.5%)
A Little Bit	10,109.0 (2.7%)	1,335.0 (0.9%)	11,551.6 (3.0%)
Somewhat	32,967.0 (8.9%)	6,371.0 (4.5%)	36,032.5 (9.5%)
Quite A Bit	72,619.0 (19.5%)	26,325.0 (18.6%)	73,812.1 (19.5%)
Extremely	233,818.0 (62.8%)	106,099.0 (74.8%)	230,213.6 (60.8%)
**Health Material Assistance**			
Skip	15,315.0 (4.1%)	909.0 (0.6%)	18,276.8 (4.8%)
Always	15,235.0 (4.1%)	2,420.0 (1.7%)	18,172.8 (4.8%)
Often	15,752.0 (4.2%)	3,302.0 (2.3%)	17,237.3 (4.6%)
Sometimes	42,741.0 (11.5%)	9,036.0 (6.4%)	45,655.8 (12.1%)
Occasionally	58,544.0 (15.7%)	23,084.0 (16.3%)	58,686.1 (15.5%)
Never	224,463.0 (60.3%)	103,127.0 (72.7%)	220,782.6 (58.3%)
**Health Information Difficulty**			
Skip	17,328.0 (4.7%)	1,357.0 (1.0%)	19,284.7 (5.1%)
Always	9,017.0 (2.4%)	1,216.0 (0.9%)	11,104.2 (2.9%)
Often	9,971.0 (2.7%)	1,569.0 (1.1%)	11,021.2 (2.9%)
Sometimes	38,120.0 (10.2%)	7,349.0 (5.2%)	42,061.4 (11.1%)
Occasionally	52,140.0 (14.0%)	19,484.0 (13.7%)	53,625.7 (14.2%)
Never	245,474.0 (66.0%)	110,903.0 (78.2%)	241,714.1 (63.8%)
**Health Literacy**			
High	313,769.0 (89.9%)	134,027.0 (96.3%)	307,565.2 (88.2%)
Low	35,298.0 (10.1%)	5,141.0 (3.7%)	41,128.6 (11.8%)
Unknown	22,983	2,710	30,117
**Health Literacy – Median (IQR)**	14.0 (12.0, 15.0)	15.0 (14.0, 15.0)	14.0 (12.0, 15.0)
Unknown	22,983	2,710	30,117
**Primary Care Provider**			
Skip	1,022.0 (0.7%)	780.0 (0.6%)	4,801.1 (1.4%)
Don’t Know	447.0 (0.3%)	326.0 (0.2%)	2,952.9 (0.9%)
No	6,919.0 (4.6%)	5,811.0 (4.4%)	19,284.4 (5.6%)
Yes	143,383.0 (94.5%)	125,001.0 (94.8%)	317,790.2 (92.2%)
Unknown	220,279	9,960	33,983
**Hypertension**			
No	91,322.0 (64.4%)	91,322.0 (64.4%)	233,579.1 (61.7%)
Skip	6,172.0 (4.4%)	6,172.0 (4.4%)	22,656.3 (6.0%)
Yes	44,384.0 (31.3%)	44,384.0 (31.3%)	122,575.9 (32.4%)
Unknown	230,172	0	0
**Coronary Artery Disease**			
No	129,389.0 (91.2%)	129,389.0 (91.2%)	341,342.4 (90.1%)
Skip	6,172.0 (4.4%)	6,172.0 (4.4%)	22,656.3 (6.0%)
Yes	6,317.0 (4.5%)	6,317.0 (4.5%)	14,812.6 (3.9%)
Unknown	230,172	0	0
**Any Cancer**			
No	103,281.0 (72.8%)	103,281.0 (72.8%)	284,666.7 (75.1%)
Skip	7,716.0 (5.4%)	7,716.0 (5.4%)	28,042.0 (7.4%)
Yes	30,881.0 (21.8%)	30,881.0 (21.8%)	66,102.6 (17.5%)
Unknown	230,172	0	0
**Skin Cancer**			
No	120,601.0 (85.0%)	120,601.0 (85.0%)	325,757.9 (86.0%)
Skip	7,716.0 (5.4%)	7,716.0 (5.4%)	28,042.0 (7.4%)
Yes	13,561.0 (9.6%)	13,561.0 (9.6%)	25,011.4 (6.6%)
Unknown	230,172	0	0
**Lung Disease**			
No	101,151.0 (71.3%)	101,151.0 (71.3%)	262,096.8 (69.2%)
Skip	6,246.0 (4.4%)	6,246.0 (4.4%)	23,721.4 (6.3%)
Yes	34,481.0 (24.3%)	34,481.0 (24.3%)	92,993.1 (24.5%)
Unknown	230,172	0	0
**Diabetes**			
No	117,793.0 (83.0%)	117,793.0 (83.0%)	296,277.0 (78.2%)
Skip	7,526.0 (5.3%)	7,526.0 (5.3%)	26,416.1 (7.0%)
Yes	16,559.0 (11.7%)	16,559.0 (11.7%)	56,118.2 (14.8%)
Unknown	230,172	0	0
**Obesity**			
No	75,937.0 (53.5%)	75,937.0 (53.5%)	185,012.4 (48.8%)
Skip	33,183.0 (23.4%)	33,183.0 (23.4%)	110,782.9 (29.2%)
Yes	32,758.0 (23.1%)	32,758.0 (23.1%)	83,016.0 (21.9%)
Unknown	230,172	0	0
**Chronic Kidney Disease**			
No	131,919.0 (93.0%)	131,919.0 (93.0%)	344,070.0 (90.8%)
Skip	6,491.0 (4.6%)	6,491.0 (4.6%)	24,187.1 (6.4%)
Yes	3,468.0 (2.4%)	3,468.0 (2.4%)	10,554.2 (2.8%)
Unknown	230,172	0	0
Weight – Median (IQR)	1 (1, 1)	1 (1, 1)	2.6 (1.7, 6.5)

*IQR* Interquartile Range

## Results

*All of Us* (version 6) included social and demographic data from 372,050 individuals, summarized in Table 1. The participants had a median age of 53 years, 59.8% were female, and 86.6% identified as straight. There were ten self-identified racial/ethnic categories, with the largest categories being White (54.0%), Black (19.9%), and Hispanic/Latino (16.7%). Incomes ranged from less than $10,000 (14.2%) to over $200,000 (6.2%), with 13% preferring not to answer. Most participants completed at least a high school degree or equivalent, with 43% of participants completing a college or advanced degree. Thirty percent of participants were insured by their employer, 17.9% by Medicaid and 15.8% by Medicare. Most participants (62.8%) reported feeling extremely confident when completing medical forms. Similarly, 60% and 66% of participants reported never needing assistance with reading health-related materials and never having difficulty understanding written health information, respectively. Nearly all participants (94.5%) who completed the Healthcare Access and Utilization instrument reported seeing a PCP within the last 12 months. The prevalence of health conditions ranged from 2.4% (chronic kidney disease) to 31.3% (hypertension) among participants who reported their health history.

**Fig. 1:**
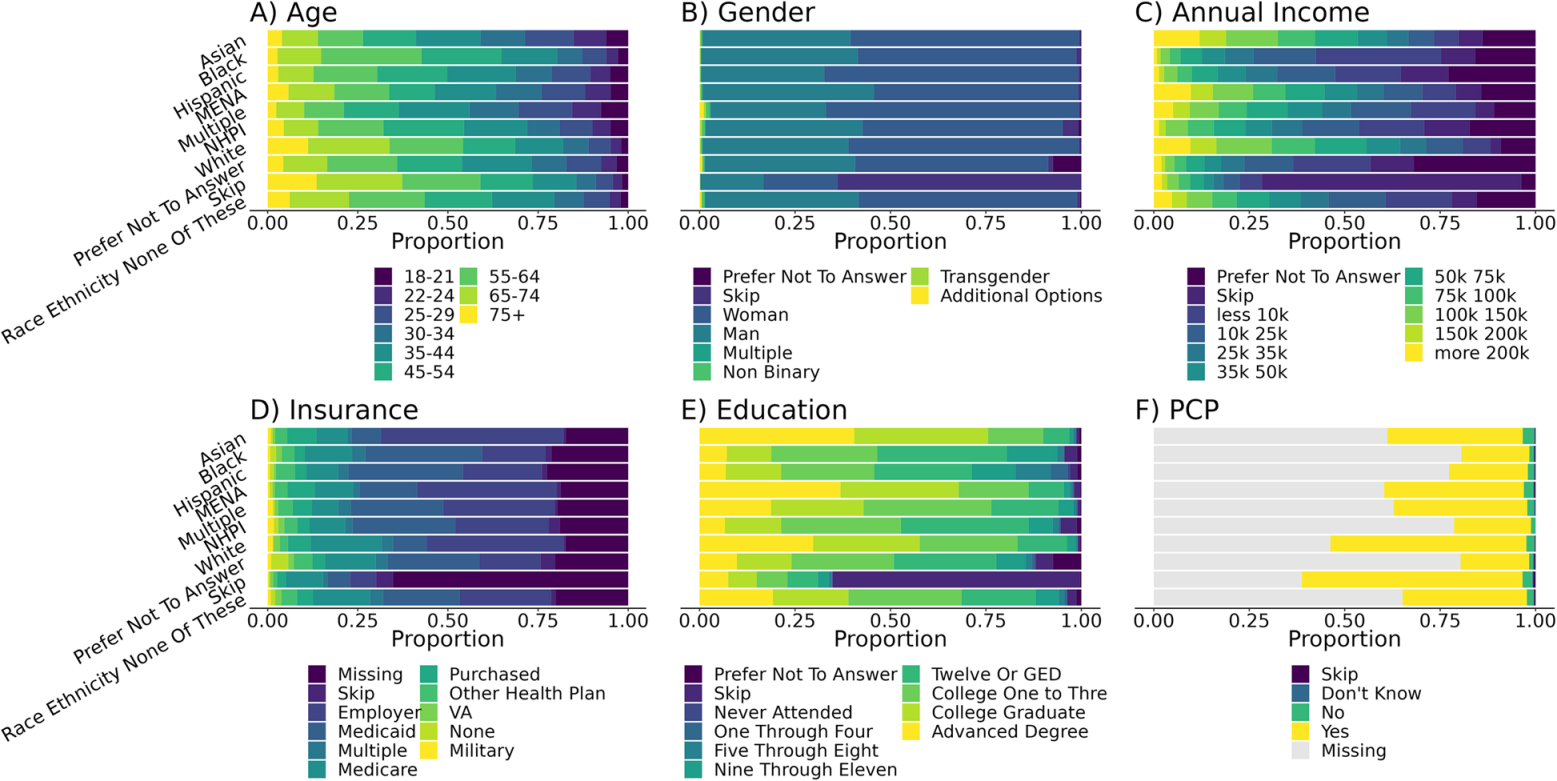
Relationship between race/ethnicity category and age, gender, annual income, health insurance, education, and PCP status. “Skip” indicates participants who filled out the corresponding *All of Us* instruments (Supplemental Tables 1.1 and 1.2) but skipped a question on the variable, and “Missing” indicates participants who did not see the instrument for any reason

Participants who identified as Asian (Middle Eastern/North African, and White were the most likely to report incomes greater than $200,000 per year (11.9%, 9.4%, 9.5%), advanced degrees (40.3%, 36.5%, 29.7%), and employer/union insurance (50.6%, 38.6%, 38.0%), whereas participants who identified as Black, Hispanic, and Native Hawaiian/Pacific Islander were the most likely to report incomes less than $10,000 per year (32.9%, 17.2%, 16.9%), less than a high school education (15.1%, 25.8%, 6.4%), and Medicaid insurance (32.3%, 31.8%, 28.1%). Participants who identified as Black, Hispanic, or Native Hawaiian/Pacific Islander were also more likely to mark “prefer not to answer”. There was substantial income, educational, and insurance diversity within each racial/ethnic category as well (Fig. 1 and Supplemental Table 2). All racial/ethnic categories self-reported high health literacy (Supplemental Fig. 1). Those who skipped the race/ethnicity question were also more likely to skip all other demographic questions. Annual household income was skipped (7.8%) or preferred not to answer (13.0%) most frequently. Racial/ethnic groups demonstrated large differences in self-reported disease prevalence (Supplemental Table 3). For example, among participants who reported clinical histories, Black participants were the most likely to report hypertension (45.1%), and Asian participants were the least likely to report obesity (9.0%).

**Fig. 2:**
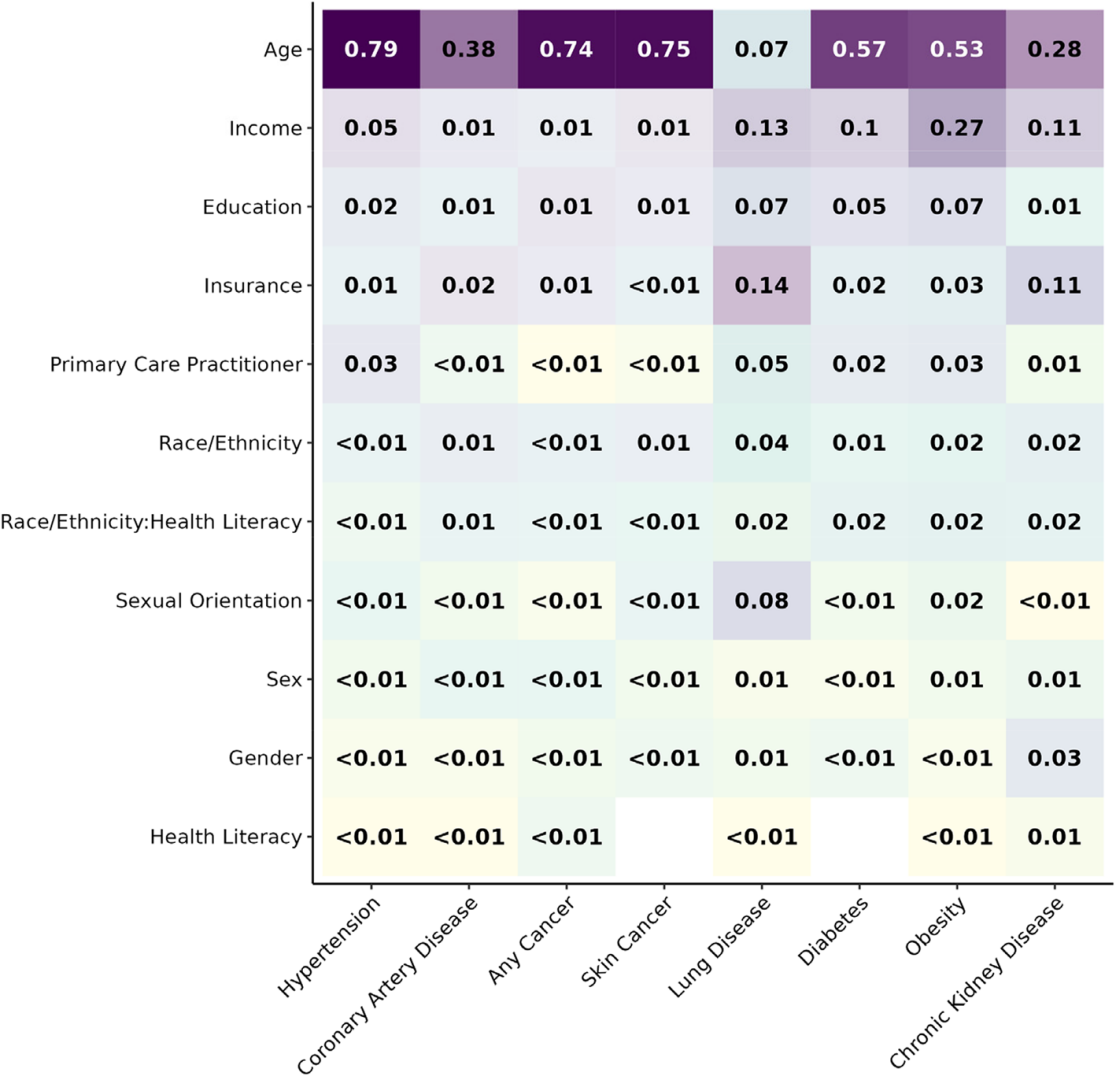
Relative importance of selected social and demographic variables on health conditions based on adequacy index. The values reflect the proportion of health condition variance (column) explained by each sociodemographic factor (row)

Inverse probability weighting generated a pseudo-population very similar to the full *All of Us* (version 6) population (Table 2 and Supplemental Fig. 2). In general, age, income, education, insurance, and race/ethnicity were the most important predictors across all assessed self-reported health conditions (Fig. 2). However, each health condition has a different order of the relative importance of each sociodemographic factor. For most health conditions, age was the most important predictor. However, age was the 5th most important predictor for a self-reported history of lung disease, which was relatively more impacted by health insurance type. We also found that income was a better predictor of self-reporting obesity compared to the other health conditions. Across most diseases, health literacy was the least predictive variable. Overall, the relative predictive value of sociodemographic factors varied greatly among chronic health conditions.

**Table 2:** Predictive model characteristics

	**Hypertension**	**Coronary Artery Disease**	**Any** **Cancer**	**Skin Cancer**	**Lung Disease**	**Diabetes**	**Obesity**	**Chronic Kidney Disease**
Sample Size (unweighted)	123983	123983	122507	122507	123818	122656	100332	123540
Sample Size (weighted)	302866	302866	298782	298782	301999	299469	232176	301159
Outcomes (unweighted)	41980	6044	29399	12936	32006	15616	30610	3295
Degrees of Freedom	77	77	77	77	77	77	77	77
Predictors per Outcome	545.19	78.49	381.81	168.00	415.66	202.81	397.53	42.79
AIC	164800.77	45098.27	125697.32	68608.24	163483.03	107876.76	137616.92	35417.84
Full Likelihood Ratio (-2*log-likelihood)	6979.78	3235.65	8083.55	5983.75	1200.33	2942.69	2527.37	984.03
Age (rcs with 5 knots)	5486.89	1243.15	6021.43	4470.81	81.64	1686.21	1343.17	277.96
Sex	8.07	13.78	10.85	5.11	7.67	3.37	15.21	10.97
Gender	5.61	4.49	6.77	6.99	7.79	10.46	8.08	30.83
Orientation	13.09	4.89	5.59	20.30	91.83	9.12	47.68	4.39
Category	24.54	25.97	40.25	37.66	46.43	20.66	48.82	18.73
Health Literacy (rcs with 5 knots)	0.53	0.88	9.97	NA	0.61	NA	0.52	5.67
Category:HealthLiteracy (no rcs)	12.75	16.25	21.31	14.25	29.05	45.41	52.27	17.69
Annual Income	318.10	47.02	41.72	84.69	151.80	280.56	673.87	112.04
Education	126.89	20.22	114.16	56.67	89.97	140.78	168.63	11.54
Insurance Type	47.34	57.35	57.33	21.06	172.16	63.02	65.97	108.56
PCP	175.38	9.06	3.89	2.72	60.20	67.47	85.84	9.65

*rcs* Restricted Cubic Spline, *NA* not applicable; “:” denotes interaction term, *PCP* primary care provider

## Discussion

In this study, we analyzed the relative contribution of sociodemographic factors to chronic diseases in a large, diverse national sample. Age was the most predictive for self-reporting each health condition. Despite evidence that health literacy is a strong predictor of chronic diseases and health care utilization [48–52], we found that health literacy was the overall weakest predictor of chronic diseases among *All of Us* participants. This may be due, in part, to the lack of response variability within this cohort or response bias inherent to self-reporting. Previous studies also suggest that the Brief Health Literacy Screen is more sensitive to identifying patients with inadequate health literacy than marginal health literacy [34]. To our knowledge, this study is the largest population in which the Brief Health Literacy Screen has been used; further studies may be necessary to understand its predictive value for chronic diseases. Overall, the differences in the relative contribution of social and demographic factors to each chronic disease underscore the importance of carefully selecting covariates when assessing disease risk and prevention. Furthermore, identifying the strongest predictors for diseases will be crucial for developing targeted interventions to prevent health disparities.

The *All of Us*participants encompass a wide range of demographic factors, social identities, and health conditions. Previous databases have been limited by a lack of diversity among study participants, leading to the exclusion of many marginalized groups in research. The UK Biobank contains data from over 500,000 individuals, 94% of whom identify as White [53, 54]. Similarly, the original Framingham Heart Study was 100% White and, despite the addition of new cohorts, 94% of current participants are White [55]. Furthermore, despite the presence of individuals with diverse backgrounds within these databases, many researchers exclude non-White races in their studies due to low sampling. *All of Us*, on the other hand, seeks to improve health research diversity by actively including participants from groups historically excluded from research, thereby strengthening its use for health disparities research [31]. Based on 2021 United States Census Bureau data, a representative sample of Americans should be about 59% non-Hispanic White [56]. In our study, we found that 54% of *All of Us* participants identified as White, which is significantly more reflective of the national population than other databases. The rich diversity and scale of *All of Us* makes it a powerful tool for studying health conditions across various social variables, including race/ethnicity.

There are several limitations to this study. The data were self-reported, which introduces measurement error that likely differs by baseline sociodemographic characteristics, and cross-sectional, which prevents any causal interpretations from our predictive models. Participants in the *All of Us* Research Program were enrolled through their health provider organization or online, which may explain the disproportionately high rate of health literacy (89.9%) and PCP status (94.5%). Furthermore, to appropriately compare predictive value of sociodemographic factors across health conditions, we did not change each model for each health condition (e.g., changing the interaction terms to fit any particular condition) or account for factors such as family history or health-related behaviors (i.e., smoking, diet, exercise) specific to each health condition. Despite the large dataset, there were a small number of some health conditions, which limited which interactions we could model. For example, while we thought the interaction between income and self-reported race/ethnicity is likely pertinent in several of the available health conditions, the fact that both were categorical variables with many categories prevented model convergence. Several of the assessed sociodemographic factors likely interact with each other, and the cumulative effect of multiple sociodemographic factors may be greater than the sum of individual sociodemographic factors. Finally, the *All of Us*database is, unfortunately, missing important covariates that likely impact health outcomes, including, but not limited to: experiences with discrimination and racism [57, 58], psychosocial stress [59, 60], environmental exposures [61], food security [62], exposures to gentrification [63, 64], and interactions with the justice system [65, 66].

## Conclusion

In this study, we characterize the differences in sociodemographic factors, and chronic diseases among racial and ethnic groups, as well as the relative predictive value of sociodemographic factors for chronic diseases, using the *All of Us* database. Our findings demonstrate that the *All of Us* Research Program is well-poised to expand the diversity of population-level health outcome research in the United States. Finally, our predictive models, although missing factors that measure structural drivers of health, highlight that social and demographic factors are differentially predictive of individual health conditions and, therefore, the importance of thoughtful model generation that considers each health condition individually. Identifying the strongest predictors for each of these diseases can also guide strategies to eliminate health disparities.

### Supplementary Information

**Additional file 1: Supplemental Figure 1.** Relationship between race/ethnicity category and sex, sexual orientation, medical form confidence, health material assistance, health information difficulty, and health literacy. **Supplemental Figure 2.** Probability of completing the Personal Medical History Instrument (propensity score) used to generate inverse probability weights. Purple bars represent participants who completed the Personal Medical History Instrument, and blue bars represent participants who did not complete this survey. **Supplemental Table 1.1.** Survey Questions and Responses. **Supplemental Table 1.2.** Health Conditions (Personal Medical History Instrument). **Supplemental Table 2.** Demographic and Social Variables Stratified by Self-Identified Race/Ethnicity. **Supplemental Table 3.** Health Conditions Stratified by Self-Identified Race/Ethnicity.

## Data Availability

The data that support the findings of this study can be accessed via the *All of Us* Researcher Workbench (https://workbench.researchallofus.org/). The data are not publicly available and cannot be made available by the corresponding author on reasonable request due to privacy or ethical restrictions. At the time of publication, access to the *All of Us* Researcher Workbench is restricted to researchers whose institution has signed a data use agreement with *All of Us* (https://www.researchallofus.org/register/).

## Abbreviations

PCP: Primary care provider
IQR: Interquartile range
