# Supplementary material for: Relative predictive value of sociodemographic factors for chronic diseases among All of Us participants: a descriptive analysis
Source: BMC Public Health. 2024 Feb 8;24:405. doi: 10.1186/s12889-024-17834-1 (PMC10851469; doi:10.1186/s12889-024-17834-1)
Supplement: Supplementary file 1 — Additional file 1: Supplemental Figure 1. Relationship between race/ethnicity category and sex, sexual orientation, medical form confidence, health material assistance, health information difficulty, and health literacy. Supplemental Figure 2. Probability of completing the Personal Medical History Instrument (propensity score) used to generate inverse probability weights. Purple bars represent participants who completed the Personal Medical History Instrument, and blue bars represent participants who did not complete this survey. Supplemental Table 1.1. Survey Questions and Responses. Supplemental Table 1.2. Health Conditions (Personal Medical History Instrument). Supplemental Table 2. Demographic and Social Variables Stratified by Self-Identified Race/Ethnicity. Supplemental Table 3. Health Conditions Stratified by Self-Identified Race/Ethnicity. [file 12889_2024_17834_MOESM1_ESM.docx]

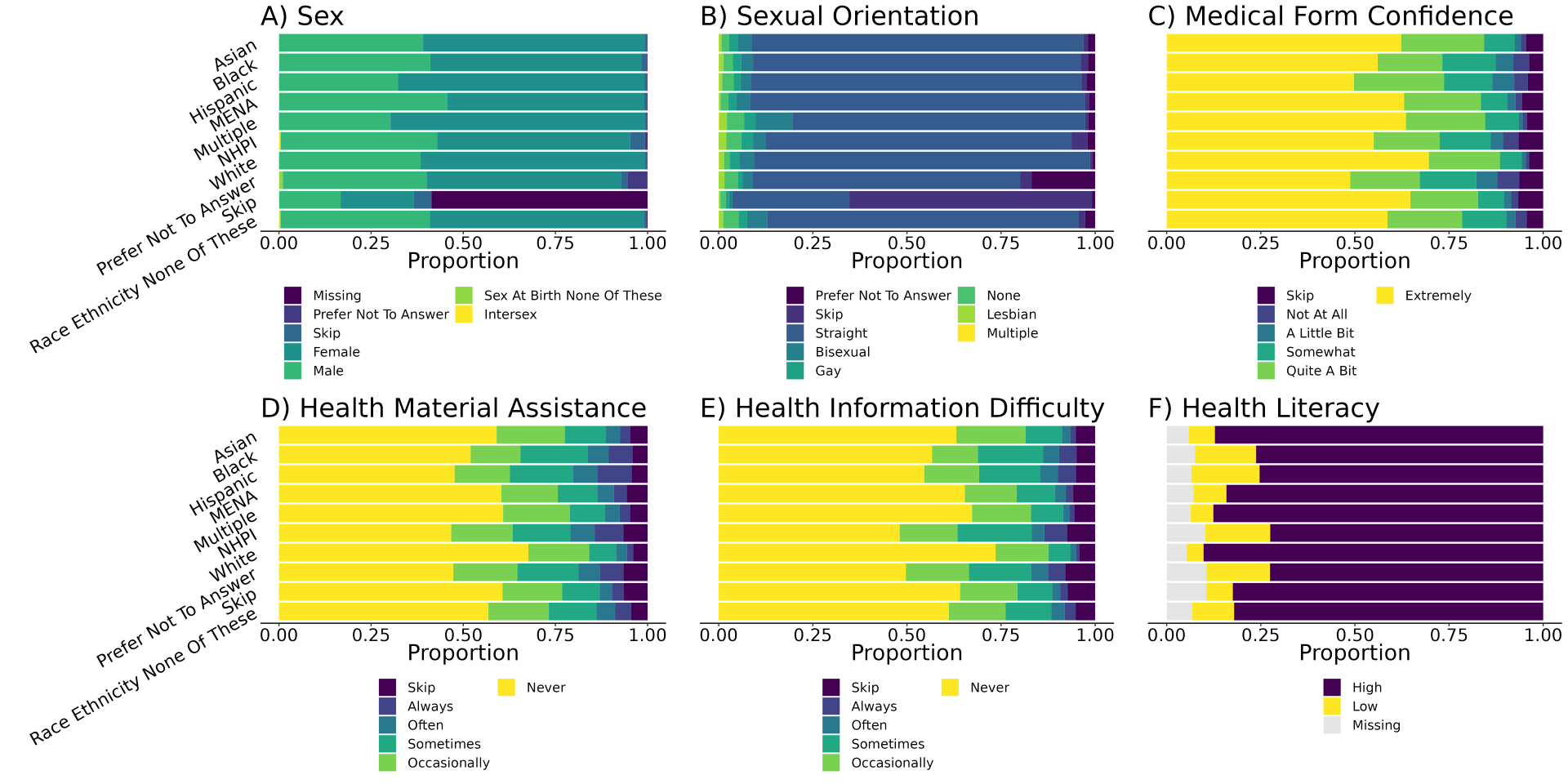


**Supplemental Figure 1.** Relationship between race/ethnicity category and sex, sexual orientation, medical form confidence, health material assistance, health information difficulty, and health literacy.


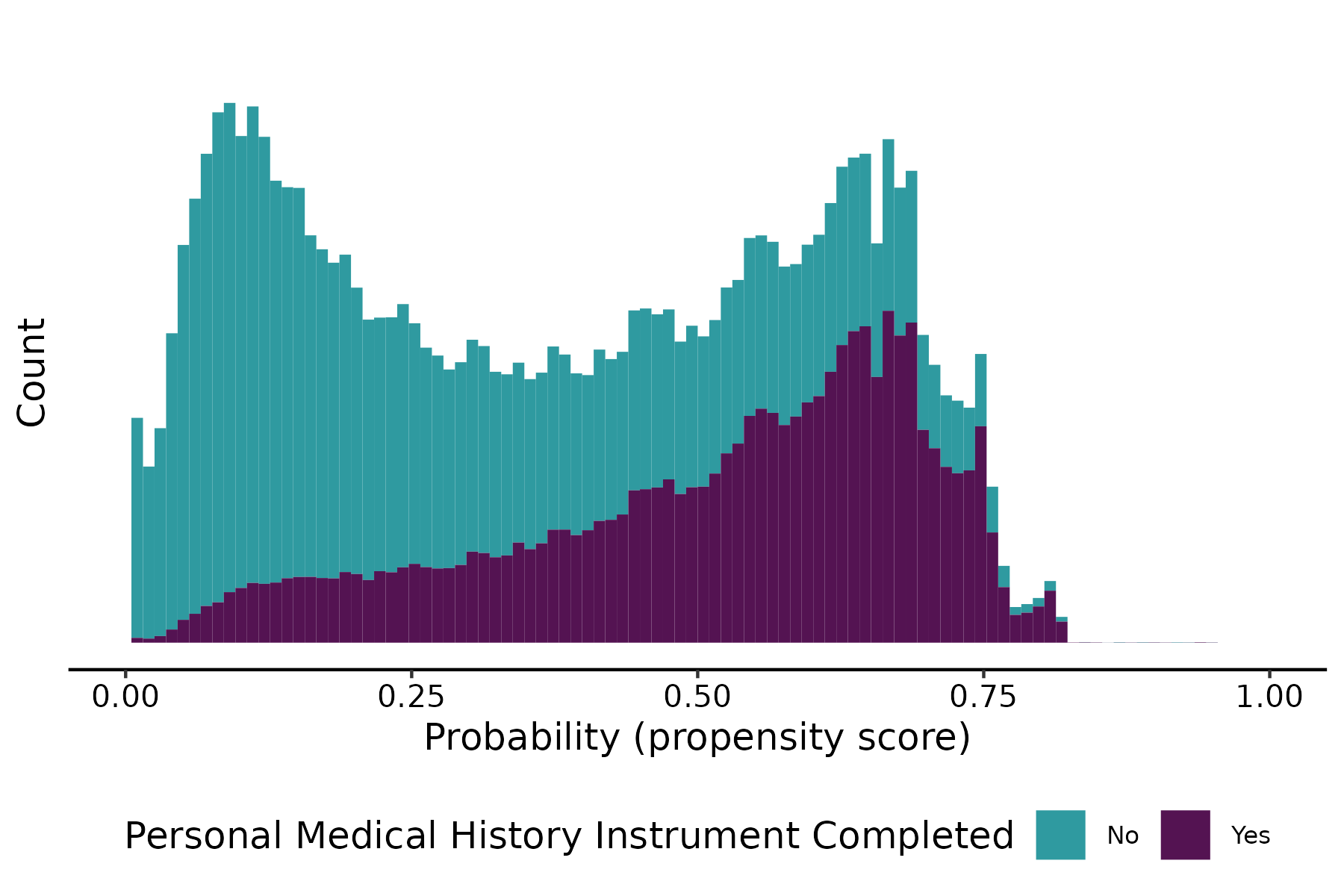
**Supplemental Figure 2.** Probability of completing the Personal Medical History Instrument (propensity score) used to generate inverse probability weights. Purple bars represent participants who completed the Personal Medical History Instrument, and blue bars represent participants who did not complete this survey.

| **Supplemental Table 1.1 Survey Questions and Responses** | | |  |
| --- | --- | --- | --- |
| **Variable** | **Instrument** | **Question(s)/Calculation** | **Answer Choices** |
| **Age** | **Calculated** | **[survey_datetime] – [date_of_birth]** |  |
| **Sex** | **Basics** | ***What was your biological sex assigned at birth?*** | Missing  Prefer Not To Answer  Skip  Female  Male  Sex At Birth None Of These  Intersex |
| **Gender** | **Basics** | ***What terms best express how you describe your gender identity?*** | Prefer Not To Answer  Skip  Woman  Man  Multiple  Non Binary  Transgender  Additional Options |
| **Sexual Orientation** | **Basics** | ***Which of the following best represents how you think of yourself?*** | Prefer Not To Answer  Skip  Straight  Bisexual  Gay  None  Lesbian  Multiple |
| **Race/ Ethnicity** | **Basics** | ***Which categories describe you?*** | Asian  Black  Hispanic  MENA  Multiple  NHPI  Prefer Not To Answer  Race Ethnicity None Of These  Skip  White |
| **Annual Income** | **Basics** | ***What is your annual household income from all sources?*** | Prefer Not To Answer  Skip  less 10k  10k 25k  25k 35k  35k 50k  50k 75k  75k 100k  100k 150k  150k 200k  more 200k |
| **Education** | **Basics** | ***What is the highest grade or year of school you completed?*** | Prefer Not To Answer  Skip  Never Attended  One Through Four  Five Through Eight  Nine Through Eleven  Twelve Or GED  College One to Three  College Graduate  Advanced Degree |
| **Health Insurance** | **Basics** | ***Are you covered by health insurance or some other kind of health care plan?*** | Missing  Skip  Employer Or Union  Medicaid  Multiple  Medicare  Purchased  Other Health Plan  VA  None  Military |
| **Medical Form Confidence** | **Overall Health** | ***How confident are you filling out medical forms by yourself?*** | Extremely [1]  Quite A Bit [2]  Somewhat [3]  A Little Bit [4]  Not At All [5]  Skip |
| **Health Material Assistance** | **Overall Health** | ***How often do you have someone help you read health-related materials?*** | Never [1]  Occasionally [2]  Sometimes [3]  Often [4]  Always [5]  Skip |
| **Health Information Difficulty** | **Overall Health** | ***How often do you have problems learning about your medical condition because of difficulty understanding written information?*** | Never [1]  Occasionally [2]  Sometimes [3]  Often [4]  Always [5]  Skip |
| **Health Literacy** | **Calculated** | **[Medical Form Confidence] + [Health Material Assistance] + [Health Information Difficulty]** |  |
| **Primary Care Provider (MD)** | **Healthcare Access & Utilization** | **During the past 12 months, have you seen or talked to any of the following health care providers about your own health? A general doctor who treats a variety of illnesses (a physician in general practice, primary care, family medicine, or internal medicine)** | Yes [1]  No [2]  Don’t Know [3]  Skip [4] |
| **Primary Care Provider (APP)** | **Healthcare Access & Utilization** | **During the past 12 months, have you seen or talked to any of the following health care providers about your own health? A nurse practitioner, physician assistant, or midwife** | Yes [1]  No [2]  Don’t Know [3]  Skip [4] |
| **Primary Care Provider** | **Calculated** | **if [MD] = 1 \| [APP] = 1, then Yes**  **elseif ([MD] = 2 & ([APP] in {2, 3, 4})) \| ([APP] = 2 & ([MD] in {2, 3, 4})), then No**  **elseif ([MD] = 3 & ([APP] in {3, 4})) \| ([APP] = 3 & ([MD] in {3, 4})), then Don’t Know**  **elseif [MD] = 4 & [APP] = 4, then Skip**  **else Missing** | Yes  No  Don’t Know  Skip  Missing |
| [ ] denotes a variable directly from the *All Of Us* database or one of our variable names when generating calculated values; & denotes a logical AND; \| denotes a logical OR; MD – medical doctor; APP – advanced practice provider | | | |

| **Supplemental Table 1.2 Health Conditions (Personal Medical History Instrument)** | | | | |
| --- | --- | --- | --- | --- |
| **Outcome** | **Question** | **Yes** | **No** | **Missing** |
| **Hypertension** | Heart and blood conditions: Has a doctor or health care provider ever told you that you have…? | Hypertension | Anemia, Atrial Fibrillation,  Bleeding Disorder,  Congestive Heart Failure, Coronary Artery, Heart Attack, Heart Valve Disease, High Cholesterol, Peripheral Vascular Disease, Pulmonary Embolism, Sickle Cell Disease, Stroke, Transient Ischemic Attack, No Heart or Blood Condition, Other Heart or Blood Condition, How Old Were You Urinary Tract: Adolescent | No matching concept,  Skip |
| **Coronary Artery Disease** | Heart and blood conditions: Has a doctor or health care provider ever told you that you have…? | Coronary Artery | Anemia, Atrial Fibrillation,  Bleeding Disorder,  Congestive Heart Failure, Heart Attack, Heart Valve Disease, High Cholesterol, Hypertension, Peripheral Vascular Disease, Pulmonary Embolism, Sickle Cell Disease, Stroke, Transient Ischemic Attack, No Heart or Blood Condition, Other Heart or Blood Condition, How Old Were You Urinary Tract: Adolescent | No matching concept,  Skip |
| **Cancer** | Cancer Conditions: Has a doctor or health care provider ever told you that you have or had any of the following cancers? | Bladder Cancer, Blood Cancer, Bone Cancer, Brain Cancer, Breast Cancer, Cervical Cancer, Colon Rectal Cancer,  Endocrine Cancer, Endometrial Cancer, Esophageal Cancer, Eye Cancer, Head Neck Cancer, Kidney Cancer, Lung Cancer, Other Cancer, Ovarian Cancer, Pancreatic Cancer,  Prostate Cancer, Skin Cancer, Stomach Cancer, Thyroid Cancer | No Cancer | Skip |
| **Skin Cancer** | Cancer Conditions: Has a doctor or health care provider ever told you that you have or had any of the following cancers? | Skin Cancer | Bladder Cancer, Blood Cancer, Bone Cancer, Brain Cancer, Breast Cancer, Cervical Cancer, Colon Rectal Cancer, Endocrine Cancer, Endometrial Cancer, Esophageal Cancer,  Eye Cancer, Head Neck Cancer, Kidney Cancer, Lung Cancer, Other Cancer, Ovarian Cancer, Pancreatic Cancer, Prostate Cancer, Stomach Cancer, Thyroid Cancer,  No Cancer | Skip |
| **Lung Disease** | Lung conditions: Has a doctor or health care provider ever told you that you have or had any of the following respiratory conditions? | Asthma, Chronic Lung, Other Lung Condition | No Lung Condition,  Sleep Apnea | Skip |
| **Diabetes** | Hormone/ endocrine conditions: Has a doctor or health care provider ever told you that you have…? | Pre Diabetes, Type 1 Diabetes, Type 2 Diabetes, Other Diabetes | No Hormone Endocrine,  Hyperthyroidism, Hypothyroidism,  Other Thyroid,  Other Hormone Endocrine | Skip |
| **Obesity** | Hormone/ endocrine conditions: Has a doctor or health care provider ever told you that you have…? | Obesity | Acne, Allergies, Endometriosis, Enlarged Prostate, Fibroids, PCOS, Reactions Anesthesia, Skin Condition, Other Diagnosis, Vitamin B Deficiency, Vitamin D Deficiency | Skip |
| **Chronic Kidney Disease** | Kidney conditions: Has a doctor or health care provider ever told you that you have…? | Kidney With Dialysis, Kidney Without Dialysis | Kidney Stones,  Acute Kidney No Dialysis, No Kidney Condition,  Other Kidney Condition | Skip |


| **Supplemental Table 2. Demographic and Social Variables Stratified by Self-Identified Race/Ethnicity** | | | | | | | | | | | | |
| --- | --- | --- | --- | --- | --- | --- | --- | --- | --- | --- | --- | --- |
|  | | | Asian | Black | Hispanic | MENA | Multiple | NHPI | White | Prefer Not to Answer | Skip | None Of These |
|  | | | N = 13,302 | N = 73,992 | N = 62,285 | N = 2,816 | N = 1,097 | N = 591 | N = 200,804 | N = 2,354 | N = 10,915 | N = 3,894 |
| **Characteristic** | | |  |  |  |  |  |  |  |  |  |  |
| Age — Median (IQR) | | | 39.0 (28.0, 55.4) | 51.8 (38.0, 60.4) | 44.4 (31.3, 57.2) | 41.6 (29.9, 60.0) | 36.9 (27.7, 52.1) | 47.3 (32.4, 59.0) | 56.8 (39.4, 68.2) | 46.8 (33.8, 59.8) | 59.2 (43.2, 69.7) | 51.3 (37.4, 63.3) |
| Sex | | |  |  |  |  |  |  |  |  |  |  |
|  | | Missing | — | 85 (0.1%) | 33 (0.1%) | — | — | — | 83 (0%) | — | 6394 (58.6%) | — |
|  | | Prefer Not to Answer | — | 49 (0.1%) | 46 (0.1%) | — | — | — | 50 (0%) | 127 (5.4%) | — | — |
|  | | Skip | 69 (0.5%) | 1082 (1.5%) | 444 (0.7%) | 23 (0.8%) | — | 23 (3.9%) | 1409 (0.7%) | 41 (1.7%) | 513 (4.7%) | 25 (0.6%) |
|  | | Female | 8010 (60.2%) | 42371 (57.3%) | 41661 (66.9%) | 1520 (54.0%) | 757 (69.0%) | 309 (52.3%) | 122164 (60.8%) | 1242 (52.8%) | 2165 (19.8%) | 2262 (58.1%) |
|  | | Male | 5200 (39.1%) | 30351 (41.0%) | 20072 (32.2%) | 1270 (45.1%) | 332 (30.3%) | 252 (42.6%) | 77030 (38.4%) | 918 (39.0%) | 1819 (16.7%) | 1573 (40.4%) |
|  | | None Of These | — | 34.0 (0%) | — | — | — | — | 39 (0%) | 23 (1.0%) | — | — |
|  | | Intersex | — | — | — | — | — | — | 29 (0%) | — | — | — |
| Gender | | |  |  |  |  |  |  |  |  |  |  |
|  | | Prefer Not to Answer | — | 84.(0.1%) | 122 (0.2%) | — | — | — | 116 (0.1%) | 175 (7.4%) | — | — |
|  | | Skip | 59 (0.4%) | 913 (1.2%) | 367 (0.6%) | — | — | 27 (4.6%) | 1193 (0.6%) | 30 (1.3%) | 6952 (63.7%) | 30 (0.8%) |
|  | | Woman | 7954 (59.8%) | 42369 (57.3%) | 41460 (66.6%) | 1503 (53.4%) | 724 (66.0%) | 308 (52.1%) | 121233 (60.4%) | 1191 (50.6%) | 2130 (19.5%) | 2226 (57.2%) |
|  | | Man | 5162 (38.8%) | 30337 (41.0%) | 20108 (32.3%) | 1264 (44.9%) | 328 (29.9%) | 244 (41.3%) | 76625 (38.2%) | 925 (39.3%) | 1797 (16.5%) | 1561 (40.1%) |
|  | | Multiple | 26 (0.2%) | 25 (0.0%) | 41 (0.1%) | — | — | — | 314 (0.2%) | — | — | — |
|  | | Non-Binary | 45 (0.3%) | 64 (0.1%) | 77 (0.1%) | — | — | — | 670 (0.3%) | — | — | 27 (0.7%) |
|  | | Transgender | 23 (0.2%) | 156 (0.2%) | 78 (0.1%) | — | — | — | 462 (0.2%) | — | — | — |
|  | | Additional Options | — | 44 (0.1%) | 32 (0.1%) | — | — | — | 191 (0.1%) | — | — | — |
| Sexual Orientation | | |  |  |  |  |  |  |  |  |  |  |
|  | | Prefer Not to Answer | 250 (1.9%) | 1388 (1.9%) | 1384 (2.2%) | 44 (1.6%) | — | — | 1275 (0.6%) | 398 (16.9%) | 94 (0.9%) | 103 (2.6%) |
|  | | Skip | 167 (1.3%) | 1376 (1.9%) | 871 (1.4%) | 31 (1.1%) | — | 26 (4.4%) | 1408 (0.7%) | 72 (3.1%) | 7027 (64.4%) | 67 (1.7%) |
|  | | Straight | 11709 (88.0%) | 64456 (87.1%) | 54697 (87.8%) | 2495 (88.6%) | 853 (77.8%) | 477 (80.7%) | 179092 (89.2%) | 1675 (71.2%) | 3394 (31.1%) | 3224 (82.8%) |
|  | | Bisexual | 498 (3.7%) | 2332 (3.2%) | 1699 (2.7%) | 101 (3.6%) | 107 (9.8%) | — | 8120 (4.0%) | 59 (2.5%) | 94 (0.9%) | 207 (5.3%) |
|  | | Gay | 311 (2.3%) | 1628 (2.2%) | 1137 (1.8%) | 62 (2.2%) | 34 (3.1%) | — | 5040 (2.5%) | 29 (1.2%) | 99 (0.9%) | 87 (2.2%) |
|  | | None | 265 (2.0%) | 1859 (2.5%) | 1912 (3.1%) | 64 (2.3%) | 50 (4.6%) | 27 (4.6%) | 3036 (1.5%) | 85 (3.6%) | 158.0 (1.4%) | 159 (4.1%) |
|  | | Lesbian | 99 (0.7%) | 939 (1.3%) | 567 (0.9%) | — | 23 (2.1%) | — | 2760 (1.4%) | 36 (1.5%) | 47 (0.4%) | 46 (1.2%) |
|  | | Multiple | — | — | — | — | — | — | 73 (0%) | — | — | — |
| Annual Income | | |  |  |  |  |  |  |  |  |  |  |
|  | | Prefer Not To Answer | 1852 (13.9%) | 11764 (15.9%) | 14202 (22.8%) | 406 (14.4%) | 119 (10.8%) | 99 (16.8%) | 18142 (9.0%) | 750 (31.9%) | 414 (3.8%) | 600 (15.4%) |
|  | | Skip | 834 (6.3%) | 6542 (8.8%) | 7805 (12.5%) | 193 (6.9%) | 54 (4.9%) | 79 (13.4%) | 5528 (2.8%) | 269 (11.4%) | 7407 (67.9%) | 254 (6.5%) |
|  | | less 10k | 843 (6.3%) | 24331 (32.9%) | 10727 (17.2%) | 257 (9.1%) | 186 (17.0%) | 100 (16.9%) | 14707 (7.3%) | 473 (20.1%) | 638 (5.8%) | 678 (17.4%) |
|  | | 10k 25k | 904 (6.8%) | 12096 (16.3%) | 9399 (15.1%) | 305 (10.8%) | 172 (15.7%) | 87 (14.7%) | 19045 (9.5%) | 305 (13.0%) | 470 (4.3%) | 581 (14.9%) |
|  | | 25k 35k | 748 (5.6%) | 5474 (7.4%) | 5186 (8.3%) | 181 (6.4%) | 82 (7.5%) | 46 (7.8%) | 13860 (6.9%) | 141 (6.0%) | 276 (2.5%) | 282 (7.2%) |
|  | | 35k 50k | 1027 (7.7%) | 4496 (6.1%) | 4579 (7.4%) | 231 (8.2%) | 99 (9.0%) | 39 (6.6%) | 17655 (8.8%) | 105 (4.5%) | 292 (2.7%) | 319 (8.2%) |
|  | | 50k 75k | 1477 (11.1%) | 4130 (5.6%) | 4180 (6.7%) | 287 (10.2%) | 118 (10.8%) | 52 (8.8%) | 27173 (13.5%) | 113 (4.8%) | 375 (3.4%) | 336 (8.6%) |
|  | | 75k 100k | 1300 (9.8%) | 2100 (2.8%) | 2343 (3.8%) | 233 (8.3%) | 81 (7.4%) | 40 (6.8%) | 22687 (11.3%) | 72 (3.1%) | 337 (3.1%) | 249 (6.4%) |
|  | | 100k 150k | 1806 (13.6%) | 1787 (2.4%) | 2172 (3.5%) | 304 (10.8%) | 83 (7.6%) | 32 (5.4%) | 29147 (14.5%) | 57 (2.4%) | 323 (3.0%) | 260 (6.7%) |
|  | | 150k 200k | 924 (6.9%) | 674 (0.9%) | 863 (1.4%) | 154 (5.5%) | 48 (4.4%) | — | 13713 (6.8%) | 23 (1.0%) | 151 (1.4%) | 150 (3.9%) |
|  | | more 200k | 1587 (11.9%) | 598 (0.8%) | 829 (1.3%) | 265 (9.4%) | 55 (5.0%) | — | 19147 (9.5%) | 46 (2.0%) | 232 (2.1%) | 185 (4.8%) |
| Education | | |  |  |  |  |  |  |  |  |  |  |
|  | | Prefer Not to Answer | 75 (0.6%) | 833 (1.1%) | 631 (1.0%) | — | — | — | 492 (0.2%) | 175 (7.4%) | 32 (0.3%) | 52 (1.3%) |
|  | | Skip | 102 (0.8%) | 2476 (3.3%) | 1179 (1.9%) | 45 (1.6%) | — | 25 (4.2%) | 1386 (0.7%) | 104 (4.4%) | 7080 (64.9%) | 91 (2.3%) |
|  | | Never Attended | — | 45 (0.1%) | 427 (0.7%) | — | — | — | — | — | — | — |
|  | | One Through Four | 39 (0.3%) | 143 (0.2%) | 2769 (4.4%) | — | — | — | 73 (0.0%) | — | — | — |
|  | | Five Through Eight | 66 (0.5%) | 1221 (1.7%) | 5708 (9.2%) | — | — | — | 947 (0.5%) | 38 (1.6%) | 83 (0.8%) | 63 (1.6%) |
|  | | Nine Through Eleven | 132 (1.0%) | 9721 (13.1%) | 7191 (11.5%) | 49 (1.7%) | 43 (3.9%) | 38 (6.4%) | 4551 (2.3%) | 182 (7.7%) | 311 (2.8%) | 232 (6.0%) |
|  | | Twelve Or GED | 907 (6.8%) | 25147 (34.0%) | 15867 (25.5%) | 273 (9.7%) | 193 (17.6%) | 201 (34.0%) | 26139 (13.0%) | 631 (26.8%) | 867 (7.9%) | 757 (19.4%) |
|  | | College One to Three | 1930 (14.5%) | 20546 (27.8%) | 15167 (24.4%) | 511 (18.1%) | 367 (33.5%) | 188 (31.8%) | 51376 (25.6%) | 631 (26.8%) | 884 (8.1%) | 1155 (29.7%) |
|  | | College Graduate | 4673 (35.1%) | 8610 (11.6%) | 9100 (14.6%) | 872 (31.0%) | 266 (24.2%) | 83 (14.0%) | 56095 (27.9%) | 337 (14.3%) | 817 (7.5%) | 769 (19.7%) |
|  | | Advanced Degree | 5362 (40.3%) | 5250 (7.1%) | 4246 (6.8%) | 1029 (36.5%) | 205 (18.7%) | 39 (6.6%) | 59726 (29.7%) | 230 (9.8%) | 816 (7.5%) | 748 (19.2%) |
| Health Insurance Type | | |  |  |  |  |  |  |  |  |  |  |
|  | | Missing | 2287 (17.2%) | 15763 (21.3%) | 14056 (22.6%) | 520 (18.5%) | 215 (19.6%) | 107 (18.1%) | 34889 (17.4%) | 477 (20.3%) | 7119 (65.2%) | 780 (20.0%) |
|  | | Skip | 107 (0.8%) | 1226 (1.7%) | 863 (1.4%) | 32 (1.1%) | — | — | 922 (0.5%) | 96 (4.1%) | 507 (4.6%) | 52 (1.3%) |
|  | | Employer Or Union | 6725 (50.6%) | 13068 (17.7%) | 13621 (21.9%) | 1087 (38.6%) | 341 (31.1%) | 147 (24.9%) | 76240 (38.0%) | 407 (17.3%) | 782 (7.2%) | 999 (25.7%) |
|  | | Medicaid | 1096 (8.2%) | 23936 (32.3%) | 19789 (31.8%) | 447 (15.9%) | 281 (25.6%) | 166 (28.1%) | 18940 (9.4%) | 601 (25.5%) | 678 (6.2%) | 824 (21.2%) |
|  | | Multiple | 152 (1.1%) | 2753 (3.7%) | 1768 (2.8%) | 41 (1.5%) | 39 (3.6%) | — | 6244 (3.1%) | 68 (2.9%) | 147 (1.3%) | 144 (3.7%) |
|  | | Medicare | 1158 (8.7%) | 9673 (13.1%) | 5453 (8.8%) | 319 (11.3%) | 83 (7.6%) | 65 (11.0%) | 39833 (19.8%) | 334 (14.2%) | 1128 (10.3%) | 618 (15.9%) |
|  | | Purchased | 1062 (8.0%) | 2098 (2.8%) | 2011 (3.2%) | 219 (7.8%) | 57 (5.2%) | 24 (4.1%) | 12472 (6.2%) | 82 (3.5%) | 266 (2.4%) | 164 (4.2%) |
|  | | Other Health Plan | 442 (3.3%) | 2582 (3.5%) | 3314 (5.3%) | 98 (3.5%) | 44 (4.0%) | 23 (3.9%) | 4620 (2.3%) | 124 (5.3%) | 121 (1.1%) | 170 (4.4%) |
|  | | VA | 88 (0.7%) | 1179 (1.6%) | 351 (0.6%) | — | — | — | 3479 (1.7%) | 36 (1.5%) | 90 (0.8%) | 64 (1.6%) |
|  | | None | 70 (0.5%) | 1205 (1.6%) | 714 (1.1%) | 24 (0.9%) | — | — | 455 (0.2%) | 112 (4.8%) | 35 (0.3%) | 47 (1.2%) |
|  | | Military | 115 (0.9%) | 509 (0.7%) | 345 (0.6%) | — | — | — | 2710 (1.3%) | — | 42 (0.4%) | 32 (0.8%) |
| Medical Form Confidence | | |  |  |  |  |  |  |  |  |  |  |
|  | | Skip | 609 (4.6%) | 2775 (3.8%) | 2515 (4.0%) | 154 (5.5%) | 47 (4.3%) | 40 (6.8%) | 7426 (3.7%) | 149 (6.3%) | 726 (6.7%) | 172 (4.4%) |
|  | | Extremely | 177 (1.3%) | 3140 (4.2%) | 2235 (3.6%) | 48 (1.7%) | — | 29 (4.9%) | 1833 (0.9%) | 137 (5.8%) | 199 (1.8%) | 113 (2.9%) |
|  | | Quite A Bit | 223 (1.7%) | 3455 (4.7%) | 3590 (5.8%) | 57 (2.0%) | — | — | 2316 (1.2%) | 132 (5.6%) | 211 (1.9%) | 96 (2.5%) |
|  | | Somewhat | 1070 (8.0%) | 10507 (14.2%) | 7977 (12.8%) | 207 (7.4%) | 97 (8.8%) | 78 (13.2%) | 11467 (5.7%) | 354 (15.0%) | 753 (6.9%) | 457 (11.7%) |
|  | | A Little Bit | 2953 (22.2%) | 12686 (17.1%) | 14933 (24.0%) | 568 (20.2%) | 231 (21.1%) | 105 (17.8%) | 37971 (18.9%) | 435 (18.5%) | 1964 (18.0%) | 773 (19.9%) |
|  | | Not At All | 8270 (62.2%) | 41429 (56.0%) | 31035 (49.8%) | 1782 (63.3%) | 697 (63.5%) | 322 (54.5%) | 139791 (69.6%) | 1147 (48.7%) | 7062 (64.7%) | 2283 (58.6%) |
| Health Material Assistance | | |  |  |  |  |  |  |  |  |  |  |
|  | | Skip | 629 (4.7%) | 3033 (4.1%) | 2649 (4.3%) | 158 (5.6%) | 52 (4.7%) | 42 (7.1%) | 7722 (3.8%) | 154 (6.5%) | 703 (6.4%) | 173 (4.4%) |
|  | | Never | 365 (2.7%) | 4791 (6.5%) | 5743 (9.2%) | 96 (3.4%) | 31 (2.8%) | 49 (8.3%) | 3497 (1.7%) | 151 (6.4%) | 341 (3.1%) | 171 (4.4%) |
|  | | Occasionally | 519 (3.9%) | 4225 (5.7%) | 4107 (6.6%) | 133 (4.7%) | 44 (4.0%) | 35 (5.9%) | 5980 (3.0%) | 137 (5.8%) | 377 (3.5%) | 195 (5.0%) |
|  | | Sometimes | 1467 (11.0%) | 13465 (18.2%) | 10701 (17.2%) | 309 (11.0%) | 104 (9.5%) | 99 (16.8%) | 14595 (7.3%) | 389 (16.5%) | 1108 (10.2%) | 504 (12.9%) |
|  | | Often | 2448 (18.4%) | 10014 (13.5%) | 9345 (15.0%) | 428 (15.2%) | 199 (18.1%) | 102 (17.3%) | 33191 (16.5%) | 410 (17.4%) | 1769 (16.2%) | 638 (16.4%) |
|  | | Always | 7874 (59.2%) | 38464 (52.0%) | 29740 (47.7%) | 1692 (60.1%) | 667 (60.8%) | 264 (44.7%) | 135819 (67.6%) | 1113 (47.3%) | 6617 (60.6%) | 2213 (56.8%) |
| Health Information Difficulty | | |  |  |  |  |  |  |  |  |  |  |
|  | | Skip | 685 (5.1%) | 3687 (5.0%) | 3148 (5.1%) | 165 (5.9%) | 60 (5.5%) | 44 (7.4%) | 8350 (4.2%) | 187 (7.9%) | 800 (7.3%) | 202 (5.2%) |
|  | | Never | 199 (1.5%) | 3404 (4.6%) | 3009 (4.8%) | 55 (2.0%) | — | 39 (6.6%) | 1866 (0.9%) | 105 (4.5%) | 213 (2.0%) | 112 (2.9%) |
|  | | Occasionally | 297 (2.2%) | 3126 (4.2%) | 2920 (4.7%) | 85 (3.0%) | — | — | 3026 (1.5%) | 109 (4.6%) | 233 (2.1%) | 137 (3.5%) |
|  | | Sometimes | 1278 (9.6%) | 12838 (17.4%) | 10082 (16.2%) | 290 (10.3%) | 94 (8.6%) | 120 (20.3%) | 11548 (5.8%) | 388 (16.5%) | 1005 (9.2%) | 477 (12.2%) |
|  | | Often | 2444 (18.4%) | 9035 (12.2%) | 9069 (14.6%) | 388 (13.8%) | 172 (15.7%) | 90 (15.2%) | 28294 (14.1%) | 395 (16.8%) | 1667 (15.3%) | 586 (15.0%) |
|  | | Always | 8399 (63.1%) | 41902 (56.6%) | 34057 (54.7%) | 1833 (65.1%) | 738 (67.3%) | 278 (47.0%) | 147720 (73.6%) | 1170 (49.7%) | 6997 (64.1%) | 2380 (61.1%) |
| Health Literacy | | |  |  |  |  |  |  |  |  |  |  |
|  | High | | 11602 (92.8%) | 56404 (82.4%) | 46975 (80.6%) | 2369 (90.6%) | 961 (93.6%) | 425 (79.9%) | 181129 (95.2%) | 1708 (81.1%) | 8999 (92.2%) | 3197 (88.1%) |
|  | Low | | 906 (7.2%) | 12013 (17.6%) | 11272 (19.4%) | 246 (9.4%) | 66 (6.4%) | 107 (20.1%) | 9093 (4.8%) | 398 (18.9%) | 765 (7.8%) | 432 (11.9%) |
|  | Unknown | | 794 | 5575 | 4038 | 201 | 70 | 59 | 10582 | 248 | 1151 | 265 |
| Health Literacy — Median (IQR) | | | 14.0 (12.0, 15.0) | 13.0 (11.0, 15.0) | 13.0 (10.0, 15.0) | 14.0 (12.0, 15.0) | 14.0 (12.0, 15.0) | 13.0 (10.0, 15.0) | 15.0 (13.0, 15.0) | 13.0 (10.2, 15.0) | 14.0 (13.0, 15.0) | 14.0 (12.0, 15.0) |
|  | Unknown | | 794 | 5575 | 4038 | 201 | 70 | 59 | 10582 | 248 | 1151 | 265 |
| Primary Care Provider | | |  |  |  |  |  |  |  |  |  |  |
|  | Skip | | 30 (0.6%) | 210 (1.5%) | 156 (1.1%) | — | — | — | 522 (0.5%) | — | 67 (1.0%) | — |
|  | Don’t Know | | — | 159 (1.1%) | 111 (0.8%) | — | — | — | 113 (0.1%) | — | 28 (0.4%) | — |
|  | No | | 401 (7.7%) | 826 (5.7%) | 1018 (7.2%) | 72 (6.5%) | — | — | 4202 (3.9%) | 25 (5.4%) | 279 (4.2%) | 70 (5.2%) |
|  | Yes | | 4752 (91.4%) | 13204 (91.7%) | 12861 (90.9%) | 1020 (92.1%) | 384 (94.1%) | 121 (93.1%) | 103030 (95.5%) | 423 (91.4%) | 6320 (94.4%) | 1268 (93.4%) |
|  | Unknown | | 8103 | 59593 | 48139 | 1708 | 689 | 461 | 92937 | 1891 | 4221 | 2537 |

| **Supplemental Table 3. Health Conditions Stratified by Self-Identified Race/Ethnicity** | | | | | | | | | | | |
| --- | --- | --- | --- | --- | --- | --- | --- | --- | --- | --- | --- |
|  | | Asian | Black | Hispanic | MENA | Multiple | NHPI | White | Prefer Not to Answer | Skip | None Of These |
|  | | N = 13,302 | N = 73,992 | N = 62,285 | N = 2,816 | N = 1,097 | N = 591 | N = 200,804 | N = 2,354 | N = 10,915 | N = 3,894 |
| Hypertension | |  |  |  |  |  |  |  |  |  |  |
|  | No | 4008.0 (78.6%) | 6223.0 (50.0%) | 9294.0 (69.7%) | 813.0 (75.5%) | 275.0 (72.8%) | 68.0 (59.6%) | 65819.0 (64.9%) | 251.0 (59.2%) | 3813.0 (59.7%) | 758.0 (61.8%) |
|  | Skip | 240.0 (4.7%) | 610.0 (4.9%) | 889.0 (6.7%) | 66.0 (6.1%) | — | — | 3889.0 (3.8%) | 37.0 (8.7%) | 355.0 (5.6%) | 69.0 (5.6%) |
|  | Yes | 850.0 (16.7%) | 5615.0 (45.1%) | 3160.0 (23.7%) | 198.0 (18.4%) | 94.0 (24.9%) | 38.0 (33.3%) | 31675.0 (31.2%) | 136.0 (32.1%) | 2219.0 (34.7%) | 399.0 (32.5%) |
|  | Unknown | 8204 | 61544 | 48942 | 1739 | 719 | 477 | 99421 | 1930 | 4528 | 2668 |
| Coronary Artery Disease | |  |  |  |  |  |  |  |  |  |  |
|  | No | 4763.0 (93.4%) | 11522.0 (92.6%) | 12224.0 (91.6%) | 970.0 (90.1%) | 362.0 (95.8%) | 100.0 (87.7%) | 92319.0 (91.1%) | 366.0 (86.3%) | 5677.0 (88.9%) | 1086.0 (88.6%) |
|  | Skip | 240.0 (4.7%) | 610.0 (4.9%) | 889.0 (6.7%) | 66.0 (6.1%) | — | — | 3889.0 (3.8%) | 37.0 (8.7%) | 355.0 (5.6%) | 69.0 (5.6%) |
|  | Yes | 95.0 (1.9%) | 316.0 (2.5%) | 230.0 (1.7%) | 41.0 (3.8%) | — | — | 5175.0 (5.1%) | 21.0 (5.0%) | 355.0 (5.6%) | 71.0 (5.8%) |
|  | Unknown | 8204 | 61544 | 48942 | 1739 | 719 | 477 | 99421 | 1930 | 4528 | 2668 |
| Any Cancer | |  |  |  |  |  |  |  |  |  |  |
|  | No | 4511.0 (88.5%) | 10230.0 (82.2%) | 11145.0 (83.5%) | 852.0 (79.1%) | 325.0 (86.0%) | 90.0 (78.9%) | 70555.0 (69.6%) | 298.0 (70.3%) | 4359.0 (68.2%) | 916.0 (74.7%) |
|  | Skip | 221.0 (4.3%) | 925.0 (7.4%) | 976.0 (7.3%) | 88.0 (8.2%) | — | — | 4913.0 (4.8%) | 42.0 (9.9%) | 442.0 (6.9%) | 87.0 (7.1%) |
|  | Yes | 366.0 (7.2%) | 1293.0 (10.4%) | 1222.0 (9.2%) | 137.0 (12.7%) | 41.0 (10.8%) | — | 25915.0 (25.6%) | 84.0 (19.8%) | 1586.0 (24.8%) | 223.0 (18.2%) |
|  | Unknown | 8204 | 61544 | 48942 | 1739 | 719 | 477 | 99421 | 1930 | 4528 | 2668 |
| Skin Cancer | |  |  |  |  |  |  |  |  |  |  |
|  | No | 4861.0 (95.4%) | 11487.0 (92.3%) | 12188.0 (91.3%) | 958.0 (89.0%) | 356.0 (94.2%) | 101.0 (88.6%) | 83997.0 (82.9%) | 357.0 (84.2%) | 5236.0 (82.0%) | 1060.0 (86.5%) |
|  | Skip | 221.0 (4.3%) | 925.0 (7.4%) | 976.0 (7.3%) | 88.0 (8.2%) | — | — | 4913.0 (4.8%) | 42.0 (9.9%) | 442.0 (6.9%) | 87.0 (7.1%) |
|  | Yes | — | 36.0 (0.3%) | 179.0 (1.3%) | 31.0 (2.9%) | — | — | 12473.0 (12.3%) | 25.0 (5.9%) | 709.0 (11.1%) | 79.0 (6.4%) |
|  | Unknown | 8204 | 61544 | 48942 | 1739 | 719 | 477 | 99421 | 1930 | 4528 | 2668 |
| Lung Disease | |  |  |  |  |  |  |  |  |  |  |
|  | No | 3972.0 (77.9%) | 8407.0 (67.5%) | 9578.0 (71.8%) | 771.0 (71.6%) | 237.0 (62.7%) | 69.0 (60.5%) | 72632.0 (71.6%) | 269.0 (63.4%) | 4439.0 (69.5%) | 777.0 (63.4%) |
|  | Skip | 200.0 (3.9%) | 723.0 (5.8%) | 940.0 (7.0%) | 77.0 (7.1%) | — | — | 3868.0 (3.8%) | 38.0 (9.0%) | 295.0 (4.6%) | 83.0 (6.8%) |
|  | Yes | 926.0 (18.2%) | 3318.0 (26.7%) | 2825.0 (21.2%) | 229.0 (21.3%) | 129.0 (34.1%) | 35.0 (30.7%) | 24883.0 (24.5%) | 117.0 (27.6%) | 1653.0 (25.9%) | 366.0 (29.9%) |
|  | Unknown | 8204 | 61544 | 48942 | 1739 | 719 | 477 | 99421 | 1930 | 4528 | 2668 |
| Diabetes | |  |  |  |  |  |  |  |  |  |  |
|  | No | 4542.0 (89.1%) | 9118.0 (73.2%) | 10479.0 (78.5%) | 888.0 (82.5%) | 317.0 (83.9%) | 78.0 (68.4%) | 85934.0 (84.8%) | 319.0 (75.2%) | 5154.0 (80.7%) | 964.0 (78.6%) |
|  | Skip | 181.0 (3.6%) | 794.0 (6.4%) | 879.0 (6.6%) | 90.0 (8.4%) | — | — | 5032.0 (5.0%) | 50.0 (11.8%) | 386.0 (6.0%) | 88.0 (7.2%) |
|  | Yes | 375.0 (7.4%) | 2536.0 (20.4%) | 1985.0 (14.9%) | 99.0 (9.2%) | 46.0 (12.2%) | 25.0 (21.9%) | 10417.0 (10.3%) | 55.0 (13.0%) | 847.0 (13.3%) | 174.0 (14.2%) |
|  | Unknown | 8204 | 61544 | 48942 | 1739 | 719 | 477 | 99421 | 1930 | 4528 | 2668 |
| Obesity | |  |  |  |  |  |  |  |  |  |  |
|  | No | 3229.0 (63.3%) | 5494.0 (44.1%) | 6036.0 (45.2%) | 611.0 (56.7%) | 210.0 (55.6%) | 52.0 (45.6%) | 56032.0 (55.3%) | 197.0 (46.5%) | 3421.0 (53.6%) | 655.0 (53.4%) |
|  | Skip | 1409.0 (27.6%) | 3481.0 (28.0%) | 4385.0 (32.9%) | 295.0 (27.4%) | 63.0 (16.7%) | 30.0 (26.3%) | 21748.0 (21.5%) | 119.0 (28.1%) | 1396.0 (21.9%) | 257.0 (21.0%) |
|  | Yes | 460.0 (9.0%) | 3473.0 (27.9%) | 2922.0 (21.9%) | 171.0 (15.9%) | 105.0 (27.8%) | 32.0 (28.1%) | 23603.0 (23.3%) | 108.0 (25.5%) | 1570.0 (24.6%) | 314.0 (25.6%) |
|  | Unknown | 8204 | 61544 | 48942 | 1739 | 719 | 477 | 99421 | 1930 | 4528 | 2668 |
| Chronic Kidney Disease | |  |  |  |  |  |  |  |  |  |  |
|  | No | 4887.0 (95.9%) | 11181.0 (89.8%) | 12227.0 (91.6%) | 988.0 (91.7%) | 365.0 (96.6%) | 104.0 (91.2%) | 94836.0 (93.5%) | 367.0 (86.6%) | 5857.0 (91.7%) | 1107.0 (90.3%) |
|  | Skip | 152.0 (3.0%) | 762.0 (6.1%) | 882.0 (6.6%) | 68.0 (6.3%) | — | — | 4145.0 (4.1%) | 43.0 (10.1%) | 346.0 (5.4%) | 80.0 (6.5%) |
|  | Yes | 59.0 (1.2%) | 505.0 (4.1%) | 234.0 (1.8%) | 21.0 (1.9%) | — | — | 2402.0 (2.4%) | — | 184.0 (2.9%) | 39.0 (3.2%) |
|  | Unknown | 8204 | 61544 | 48942 | 1739 | 719 | 477 | 99421 | 1930 | 4528 | 2668 |
